# Biocompatibility and Efficacy of a Linearly Cross-Linked Sodium Hyaluronic Acid Hydrogel as a Retinal Patch in Rhegmatogenous Retinal Detachment Repairment

**DOI:** 10.3389/fbioe.2022.914675

**Published:** 2022-07-04

**Authors:** Chuanzhen Zheng, Hongwei Xi, Dejia Wen, Yifeng Ke, Xiaomin Zhang, Xinjun Ren, Xiaorong Li

**Affiliations:** ^1^ Tianjin Key Laboratory of Retinal Functions and Diseases, Tianjin International Joint Research and Development Centre of Ophthalmology and Vision Science, Eye Institute and School of Optometry, Tianjin Medical University Eye Hospital, Tianjin, China; ^2^ Qisheng Biological Preparation Co., Ltd., Shanghai, China

**Keywords:** linearly cross-linked sodium hyaluronic acid hydrogel, rhegmatogenous retinal detachment, biocompatibility, efficacy, cohesiveness, viscosity

## Abstract

To prevent the migration of retinal pigment epithelium (RPE) cells into the vitreous cavity through retinal breaks after the pars plana vitrectomy for the repair of rhegmatogenous retinal detachment (RRD), sealing retinal breaks with an appropriate material appears to be a logical approach. According to a review of ocular experiments or clinical trials, the procedure for covering retinal breaks with adhesives is complex. A commercially available cross-linked sodium hyaluronic acid (HA) hydrogel (Healaflow^®^) with the injectable property was demonstrated to be a perfect retinal patch in RRD clinical trials by our team. Based on the properties of Healaflow^®^, a linearly cross-linked sodium HA hydrogel (HA-engineered hydrogel) (Qisheng Biological Preparation Co. Ltd. Shanghai, China) with the injectable property was designed, whose cross-linker and cross-linking method was improved. The purpose of this study is to report the characteristics of an HA-engineered hydrogel using Healaflow^®^ as a reference, and the biocompatibility and efficacy of the HA-engineered hydrogel as a retinal patch in the rabbit RRD model. The HA-engineered hydrogel exhibited similar dynamic viscosity and cohesiveness and G′ compared with Healaflow^®^. The G′ of the HA-engineered hydrogel varied from 80 to 160 Pa at 2% strain under 25°C, and remained constantly higher than G″ over the range of frequency from 0.1 to 10 Hz. In the animal experiment, clinical examinations, electroretinograms, and histology suggested no adverse effects of the HA-engineered hydrogel on retinal function and morphology, confirming its favorable biocompatibility. Simultaneously, our results demonstrated the efficacy of the HA-engineered hydrogel as a retinal patch in the RRD model of rabbit eyes, which can aid in the complete reattachment of the retina without the need for expansile gas or silicone oil endotamponade. The HA-engineered hydrogel could play the role of an ophthalmologic sealant due to its high viscosity and cohesiveness. This pilot study of a small series of RRD models with a short-term follow-up provides preliminary evidence to support the favorable biocompatibility and efficacy of the HA-engineered hydrogel as a promising retinal patch for sealing retinal breaks in retinal detachment repair. More cases and longer follow-up studies are needed to assess its safety and long-term effects.

## Introduction

**FIGURE 1:**
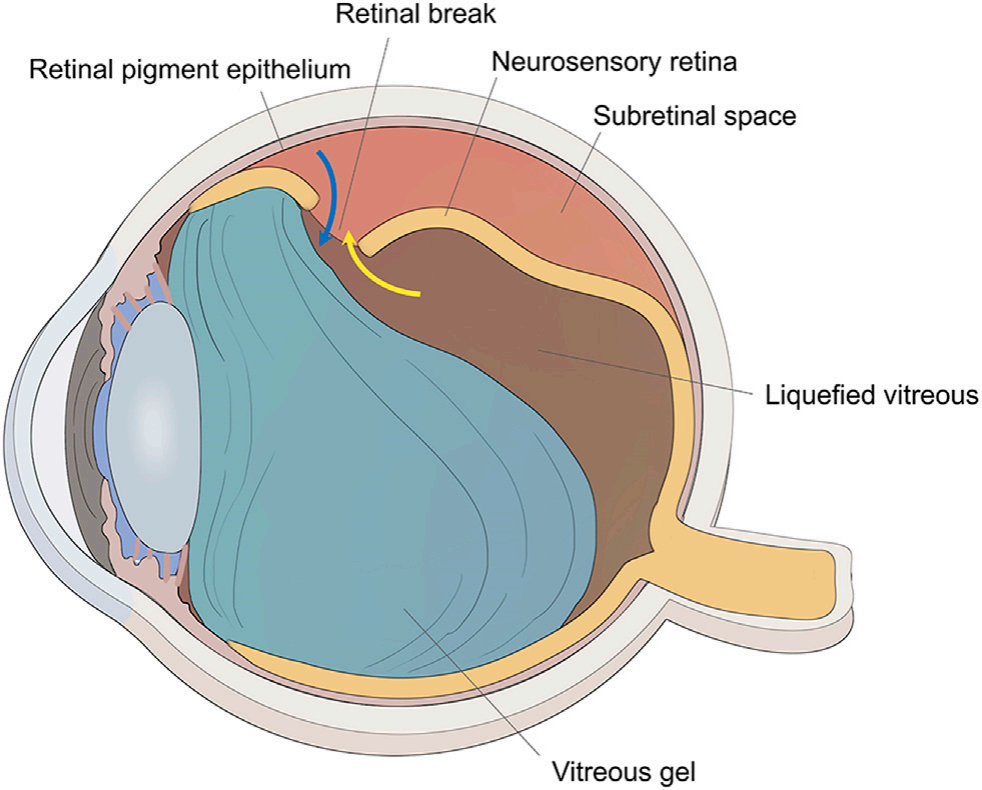
The schematic diagram of RRD. The traction of vitreous gel onto the retina created the retinal break, and liquefied vitreous penetrated the subretinal space through the retinal break to induce retinal detachment (yellow arrow). Retinal pigment epithelial cells could migrate to the vitreous cavity through the retinal break (blue arrow).

The vitreous gel is a transparent ocular tissue located between the lens and the retina, composed of 98–99% water and a framework of collagen fibers and hyaluronic acid (HA). Aging leads to vitreous liquefaction and detachment of the vitreous from the retinal surface, which may induce retinal breaks. The liquefied vitreous can pass through these breaks and accumulate in the subretinal space between the neurosensory retina and the retinal pigment epithelium (RPE) leading to a rhegmatogenous retinal detachment (RRD) (Figure 1) (Feltgen and Walter, 2014; Lumi et al., 2015). Attachment of the retina is mandatory for its proper functioning. The principles for managing RRD include treating all retinal breaks and weakening or eliminating vitreous traction using one or more of the following surgical techniques: pneumatic retinopexy, scleral buckling, and pars plana vitrectomy (PPV) (Kuhn and Aylward, 2014). PPV involves surgical removal of the vitreous, thus releasing vitreoretinal traction, and subsequent filling of the vitreous cavity with long-lasting substitutes (expansile gas or silicon oil) to prevent the connection between the subretinal space and vitreous cavity through the break; moreover, laser photocoagulation is used to build chorioretinal adhesion around the retinal breaks, and the surface tension of vitreous substitutes keeps the neurosensory retina attached to the RPE until the chorioretinal adhesion becoming sufficiently strong to seal the retinal edge around the breaks; last, after the expansile gas has been absorbed in 2 weeks–2 months or the silicon oil has been removed by a second surgery (Wagenfeld et al., 2010; Chen et al., 2015; Kontos et al., 2017; Raczynska et al., 2018; Tetsumoto et al., 2020), the vitreous cavity is filled with aqueous humor produced by the eyeball itself, which has a refractive index approximately identical to natural vitreous (Popovic et al., 2022). With the advancement of the microinvasion system, PPV combined with expansile gas or silicon oil endotamponade is gaining popularity as a first-line procedure for the repair of RRD, but the first retinal reattachment rates range from 74 to 96.3% (Bourla et al., 2010; Oshima et al., 2010; Kobashi et al., 2014; Haugstad et al., 2017; Romano et al., 2017). Proliferative vitreoretinopathy (PVR) is the major cause of surgical failure in retinal detachment, in which RPE cells migrate to the vitreous cavity through retinal breaks (Figure 1) and undergo cellular proliferation and epithelial–mesenchymal transition (EMT), resulting in the formation of a proliferative membrane on the retinal surface, whose contraction property causes a tractional retinal detachment (Pastor et al., 2016). Hence, sealing retinal breaks with an appropriate material appears to be a logical approach to prevent migration, proliferation, and EMT of RPE cells, thereby preventing PVR.

Currently, there are two major categories of clinical adhesives: synthetic glues (such as cyanoacrylate, polyethylene glycol (PEG) derivatives, and HA derivatives) and biological glues (such as fibrin). However, as a retinal patch, each has its own set of limitations, such as potential ocular toxicity, difficulty in intraocular delivery, poor adhesive force, inflammatory response, and granulomatous tissue reaction. For these and other reasons, the use of glue in the treatment of retinal detachment has not yet become a standard procedure (Hoshi et al., 2015). Nevertheless, a commercially available cross-linked sodium HA hydrogel (Healaflow^®^) (Anteis S.A., Plan Les Ouates, Switzerland) has been shown to be an ideal retinal patch. Cellular experiments and animal studies have demonstrated its favorable biocompatibility, strong and durable adhesion (Barth et al., 2014; Barth et al., 2016; Barth et al., 2019). A clinical trial conducted by our team also confirmed its strong and durable adhesion, safety, and efficacy; ease of acquisition, preservation, and delivery to the retina; and simple procedure of covering retinal breaks (Ren et al., 2020). Based on the properties of Healaflow^®^, a linearly cross-linked sodium HA hydrogel (HA-engineered hydrogel) (Qisheng Biological Preparation Co. Ltd. Shanghai, China) was designed, in which both the cross-linker and cross-linking methods were improved. The HA-engineered hydrogel was developed using the patented technology, which utilizes the fluid characteristics of HA and guides the molecular structural rearrangement of cross-linked HA by providing a certain direction of stress (Wei et al., 2019). Compared with the most commercially available cross-linked HA products, this patented technology does not include fragmentation or the sieving process (Wongprasert et al., 2022), thus protecting the molecular structure’s integrity and improving the resistance to gel degradation effectively (Wei et al., 2019). The resulting HA-engineered hydrogel was homogeneous and monophasic, showing oriented-arranged morphology. It possessed good viscoelasticity, injectability, and cohesion, which benefits *in vivo* tissue integration post-implantation (Tran et al., 2014). Different from Healaflow^®^ which uses 1, 4-butanediol diglycidyl ether (BDDE) as the cross-linker, the HA-engineered hydrogel was cross-linked by divinylsulfone (DVS). Both BDDE and DVS are the well-acknowledged industry-standard cross-linkers of the market-leading cross-linked HA products, whose stability and metabolism have been studied in detail, and their long-term safety has been verified by numerous studies and clinical practice over the years (Pérez et al., 2021). The purpose of this study is to report the characteristics of the HA-engineered hydrogel using Healaflow^®^ as a reference, and the biocompatibility and efficacy of the HA-engineered hydrogel as a retinal patch in the rabbit RRD model.

## Materials and Methods

### HA-Engineered Hydrogel

**FIGURE 2:**
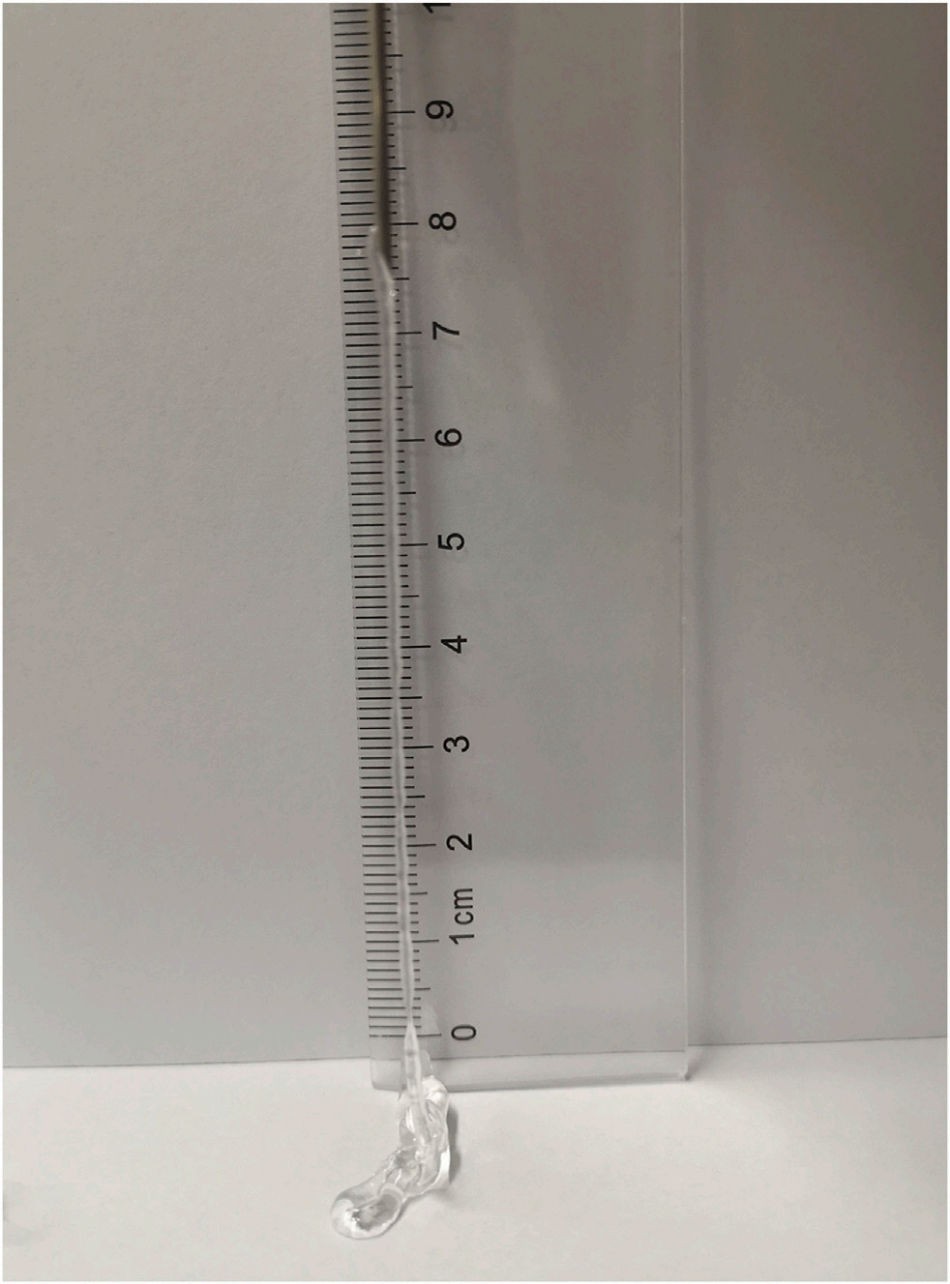
The photograph of the HA-engineered hydrogel extruded from the syringes through an 18-gauge cannula.

The HA-engineered hydrogel is a commercially available transplant, a smooth and cohesive hydrogel manufactured by Shanghai Qisheng Biological Preparation Co., Ltd. (Figure 2). It is composed of balanced salt solution (BSS), phosphate and NaCl salts, and non-animal sodium HA (16 mg/ml) that has been linearly cross-linked with DVS. Whereas the concentration of HA is 22.5 mg/ml and the cross-linker is BDDE in Healaflow^®^. The improved process of linear cross-linking in the HA-engineered hydrogel makes the sodium HA molecules exhibit linear tropism that could prolong the structural integrity. The properties of the HA-engineered hydrogel are similar to those of Healaflow^®^ (Barth et al., 2014). Table 1 summarizes their physical and chemical properties.

**TABLE 1:** Comparison of physicochemical properties between the HA-engineered hydrogel and Healaflow®.

Properties	HA-engineered hydrogel (Qisheng Biological Preparation Co. Ltd. Shanghai, China)	Healaflow® (Anteis S.A., Plan Les Ouates, Switzerland)
Cross-linker	DVS	BDDE
HA concentration	16 mg/ml	22.5 mg/ml
pH	7.3	7.0
Osmolarity	300 mOsm/kg	305 mOsm/kg
Specific gravity	1.01	1.03
Refractive index	1.341	1.341
Dynamic viscosity	252170 mPa·s	258000 mPa s

Table footer. HA, hyaluronic acid; DVS, divinyl sulfone; BDDE, 1, 4-butanediol diglycidyl ether

### Comparison of Characteristics Between HA-Engineered Hydrogel and Healaflow^®^

#### Rheology

The rheological properties of HA-engineered hydrogel and Healaflow^®^ were measured using a rheometer (Thermo Scientific HAAKE MARS III) equipped with a P35 TiL measuring geometry with a gap of 1 mm at 25°C. The applied strain was 2% and the frequency sweep was from 0.1 to 10 Hz. The storage modulus (G′) and the loss modulus (G″) of the HA-engineered hydrogel were recorded.

#### Cohesiveness

We tested the cohesiveness of HA-engineered hydrogel and Healaflow^®^ by two previously published methods. 1) The drop-weight method (Edsman et al., 2015). Three samples in each group were loaded into 1-ml glass syringes with an 18-gauge cannula and centrifugated to eliminate air bubbles. Then the samples were extruded using a mechanical testing instrument (GOTECH, China) at a constant speed. While the stress was stable, 10 drops of each sample were collected and weighed, and the average drop weight was calculated. 2) The stained hydrogel dispersion method (Edsman et al., 2015). The 1-ml gels were first stained with 30 μL of 1% toluidine blue solution between two syringes for 3 min. The gel with air removed by centrifugation was then filled into a 1-ml BD glass syringe (Becton, Dickinson and Company, Franklin Lakes, NJ, United States). Three 1000-ml beakers filled with 700 ml water were placed on a magnetic stirrer with a magnetic stirring bar at 170 revolutions per minute. The stained gel in the syringe was placed with the orifice 2 cm above the surface of the water and pushed out of the syringe at a speed of 400 mm/min with Zwick BTC-FR 2.5 TH.D09 (ZwickRoell, Ulm, Germany). Photographs were taken at 15, 75, and 90 s after the gel hit the surface. We also tested the cohesiveness of two other HA-based hydrogels, Matrifill^®^ and Janlane^®^, which were cross-linked by DVS and BDDE, separately.

### Animal Preparation

The left eyes of 24 pigmented rabbits (Dutch, weighing 2.0–3.0 kg) were tested in our study. The biocompatibility of retinal covering with the HA-engineered hydrogel was tested in 8 rabbits, and the efficacy of the HA-engineered hydrogel was tested in the remaining 16 rabbits, which were divided into two groups of 8 each: the RRD-hydrogel group and the RRD group. We certify that all applicable institutional and governmental regulations concerning the ethical use of animals were followed during this research. All procedures were performed in the left eyes of rabbits using sterile techniques. The rabbits were anesthetized with an intravenous injection of 5.0 ml midazolam (1 mg/ml) and an intramuscular injection of 1.0 ml xylazine hydrochloride (10 mg/ml). Topical anesthesia (0.4% oxybuprocaine hydrochloride drops) was also administered to the eyes. The pupils were dilated using tropicamide phenylephrine eye drops.

### Biocompatibility of HA-Engineered Hydrogel Onto Retina

#### Vitrectomy and Application of HA-Engineered Hydrogel

The conjunctiva was prepared using a 5% povidone-iodine solution. Using three ports 1 mm from the corneoscleral limbus, 25-gauge PPV was performed in all study rabbits using a Constellation system (Alcon Laboratories, Inc., Fort Worth, TX, United States) by an experienced vitreoretinal surgeon (X.R.); one port was used for the infusion cannula, while the other two ports were used for the vitreous cutter and endoilluminator optical fiber. Sclerotomy was performed *via* biplanar entry using a trocar and cannula, initially tangential to the sclera and then perpendicular, to create a self-sealing incision. The lens was not removed. Core vitrectomy was performed under a surgical microscope with a fundus wide-angle viewing system (Volk Mini Quad XL; Volk Optical, Inc., Mentor, OH, United States), and posterior vitreous detachment (PVD) was created using triamcinolone acetonide. Fluid–air exchange was performed, and 0.1 ml HA-engineered hydrogel was applied with a 27-gauge needle through a trocar and cannula to cover the retina at 2 disc diameters (DD) below the optic disc. The microcannulas were removed after vitrectomy, and the sclerotized area was gently massaged with a cotton-tipped applicator to prevent leakage. The surgical eyes received eye drops containing antibiotics and dexamethasone for 1 week after surgery.

#### Clinical Examination

Slit lamp microscopy, indirect ophthalmoscopy, and iCare tonometer were used with the pupils dilated preoperatively, and at 1, 3, and 5 days; 1 and 2 weeks; and 1 month postoperatively. Intraocular pressure (IOP) was assessed using repeated measures analysis of variance.

#### Electroretinogram

Under general and topical anesthesia and with the pupils dilated, an electroretinogram (ERG) was recorded before and 1 month after the application of the HA-engineered hydrogel. Contact lens electrodes (ChongQing IRC Medical Equipment Co., Ltd., Chongqing, China) were placed on the corneas of both eyes; a needle electrode was attached to the occipital region at the midpoint of the two ears, and a ground electrode was attached to the tail. The ERG was recorded from both eyes simultaneously using an ERG recording system (RetiMINER-C; ChongQing IRC Medical Equipment Co., Ltd.). This apparatus combines a stimulus instrument, an amplifier, and a recorder. The frequency band ranges from 0.3 to 300 Hz. The luminance values of the stimuli were 0.01, 3.0, and 30 cds/m^2^. The recording started with the weakest stimulus after 30 min of dark adaptation. No background light was applied. The light stimulus interval was 30 s, and three responses were averaged for each eye. Amplitude and implicit times were recorded for both eyes. The differences between the pre- and post-operation in the left eyes were analyzed.

#### Histology

The rabbits were killed with an overdose of pentobarbital 1 month after vitrectomy, and their eyes were enucleated for histological analysis. After enucleation, all eyes were fixed in 2% paraformaldehyde and 2.5% glutaraldehyde solution, dehydrated in a series of increasing alcohol concentrations, and embedded in paraffin. Sections cut at a thickness of 4 μm were stained with hematoxylin and eosin (H&E) and examined under a light microscope.

#### Immunofluorescence

Sections of samples embedded in paraffin were deparaffinized in a xylene ethanol series, placed in Tris-EDTA buffer for antigen retrieval (10 mM Tris, 1 mM EDTA, 0.05% Tween, pH = 9.0), and then blocked in 5% bovine serum albumin. Sections were immunostained for rhodopsin (rod photoreceptors) (Ab 5417; Abcam, United Kingdom; diluted 1:100). Detection of the primary antibodies was performed using fluorescein isothiocyanate (FITC)–conjugated goat anti-mouse IgG secondary antibody (F-2761; Thermo Fisher Scientific, United States; diluted 1:400). Nuclei were detected using 4′,6′-diamino-2-phenyl inodole (DAPI), which was included in the mounting solution (Solarbio; Beijing, China).

### Efficacy of HA-Engineered Hydrogel as a Retinal Patch in RRD Repairment

#### RRD Model

**FIGURE 3:**
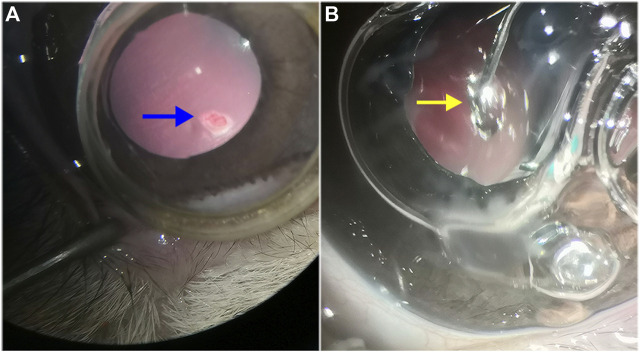
Retinal detachment and HA-engineered hydrogel coverage. **(A)** After vitrectomy and PVD in the rabbit eye, the vitreous cutter was used to make a retinal break that was approximately 1/2 DD in size (blue arrow). The break was made 2 DD inferior to the optic disc. **(B)** The HA-engineered hydrogel (yellow arrow) was gently applied over the retinal break with a 25-gauge syringe through a trocar cannula in the RRD-hydrogel group.

Complete vitrectomy and PVD were performed as previously described, and a retinal break, approximately 1/2 DD in size, was created 2 DD inferior to the optic disc using the vitreous cutter. BSS was gently infused into the subretinal space through the retinal break to create a localized retinal detachment, which was approximately 2 DD in size. Fluid–air exchange was performed to reattach the retina. In the RRD-hydrogel group, the HA-engineered hydrogel was applied with a 25-gauge syringe through a trocar and cannula to completely cover the retinal break (Figure 3). The RRD group underwent the same procedures as the RRD-hydrogel group, except for the HA-engineered hydrogel application. The microcannulas were removed after vitrectomy, and the sclerotized area was gently massaged with a cotton-tipped applicator to prevent leakage. The surgical eyes received eye drops containing antibiotics and dexamethasone for 1 week after surgery.

#### Clinical Examination

Slit lamp microscopy, indirect ophthalmoscopy, and iCare tonometer were used with the pupils dilated preoperatively, and at 1 and 7 days, and 1 and 3 months postoperatively. B-mode ultrasound and fundus photography were used to evaluate the state of the retina in both groups before surgery and at 7 days and 1 and 3 months after surgery.

### Statistical Analysis

Descriptive data were shown as mean ± standard deviation, and the difference between groups or pre- and post-operation was analyzed with paired *t*-tests or repeated measures analysis of variance. Qualitative data were presented as numbers and percentages, and were compared with the chi-square test (or Fischer’s exact test if the criteria for the chi-square test were not fulfilled). *p* < 0.05 indicated that the difference was statistically significant.

## Results

### The Physicochemical Properties of HA-Engineered Hydrogel Were Similar With Those of Healaflow^®^

#### Rheology

**FIGURE 4:**
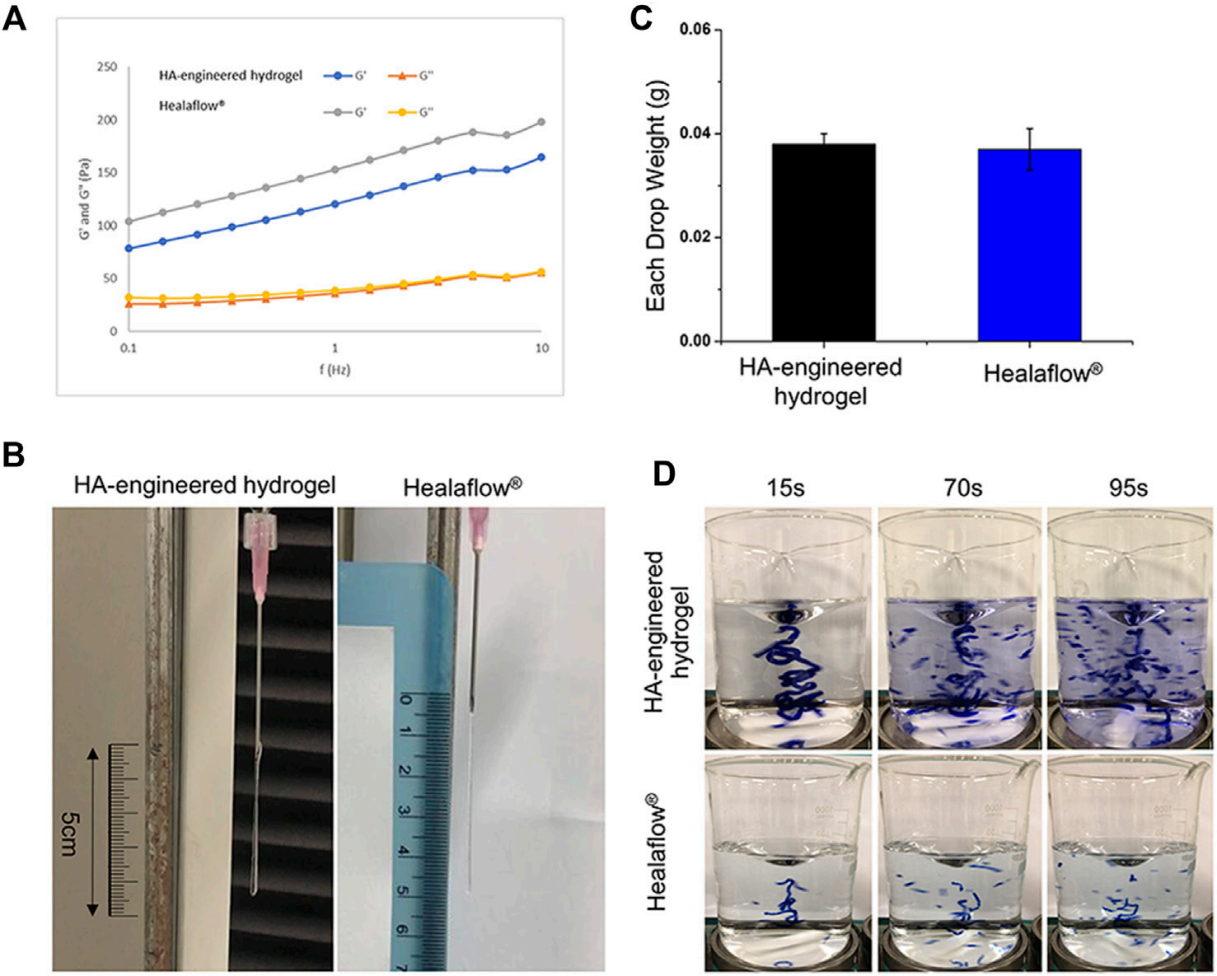
Comparison of characteristics between HA-engineered hydrogel and Healaflow^®^. **(A)** G′ and G″ of HA-engineered hydrogel and Healaflow^®^ at 2% strain over the frequency sweep from 0.1 to 10 Hz under 25°C. **(B,C)** Cohesiveness characterization of HA-engineered hydrogel and Healaflow^®^ using the drop-weight method. **(B)** The photographs of HA-engineered hydrogel and Healaflow^®^ when they were about to break. **(C)** The average drop weight of the collected HA-engineered hydrogel and Healaflow^®^ hydrogels (*n* = 3). **(D)** Cohesiveness characterization of HA-engineered hydrogel and Healaflow^®^ using the stained hydrogel dispersion method. The images were captured at the time point of 15, 75, and 90 s after the gel hit the surface.

The HA-engineered hydrogel sealant and Healaflow^®^ exhibited a higher G′ than G″ over a wide range of frequency from 0.1 to 10 Hz (Figure 4A), indicating that both the two hydrogels were gel-like rather than the sol-like in nature. The G’ of the HA-engineered hydrogel varied from 80 to 160 Pa at 2% strain under 25°C, which was similar to that of Healaflow^®^ (100–190 Pa). In addition, as shown in Table 1, the dynamic viscosity was 252,170 mPa s at 0.25 Hz under 25°C, which was similar to that of Healaflow^®^ (258,000 mPa s).

#### Cohesiveness

As shown in Figure 4B, the stretched height of the HA-engineered hydrogel gel extruded from the syringe was about 4.5 cm, which was similar to that of the Healaflow^®^ sample. The drop weight of the HA-engineered hydrogel was 0.038 ± 0.002 g, which was also similar to that of the Healaflow^®^ sample (0.037 ± 0.004 g) (Figure 4C).

As shown in Figure 4D, both HA-engineered hydrogel and Healaflow^®^ remained intact at the time point of 15 s, while dispersed into obvious small strips gradually afterward. By comparison, the Matrifill^®^ and Janlane^®^ samples dispersed quickly once stirred in the water, and scattered into barely visible particles (Supplementary Figure S1). The dispersion degree of HA-engineered hydrogel and Healaflow^®^ was much lower than that of Matrifill^®^ and Janlane^®^.

The aforementioned results indicated that the cohesiveness of the HA-engineered hydrogel was similar to that of Healaflow^®^ and much higher than those of Matrifill^®^ and Janlane^®^.

### HA-Engineered Hydrogel Showed Favorable Biocompatibility With Retina

**FIGURE 5:**
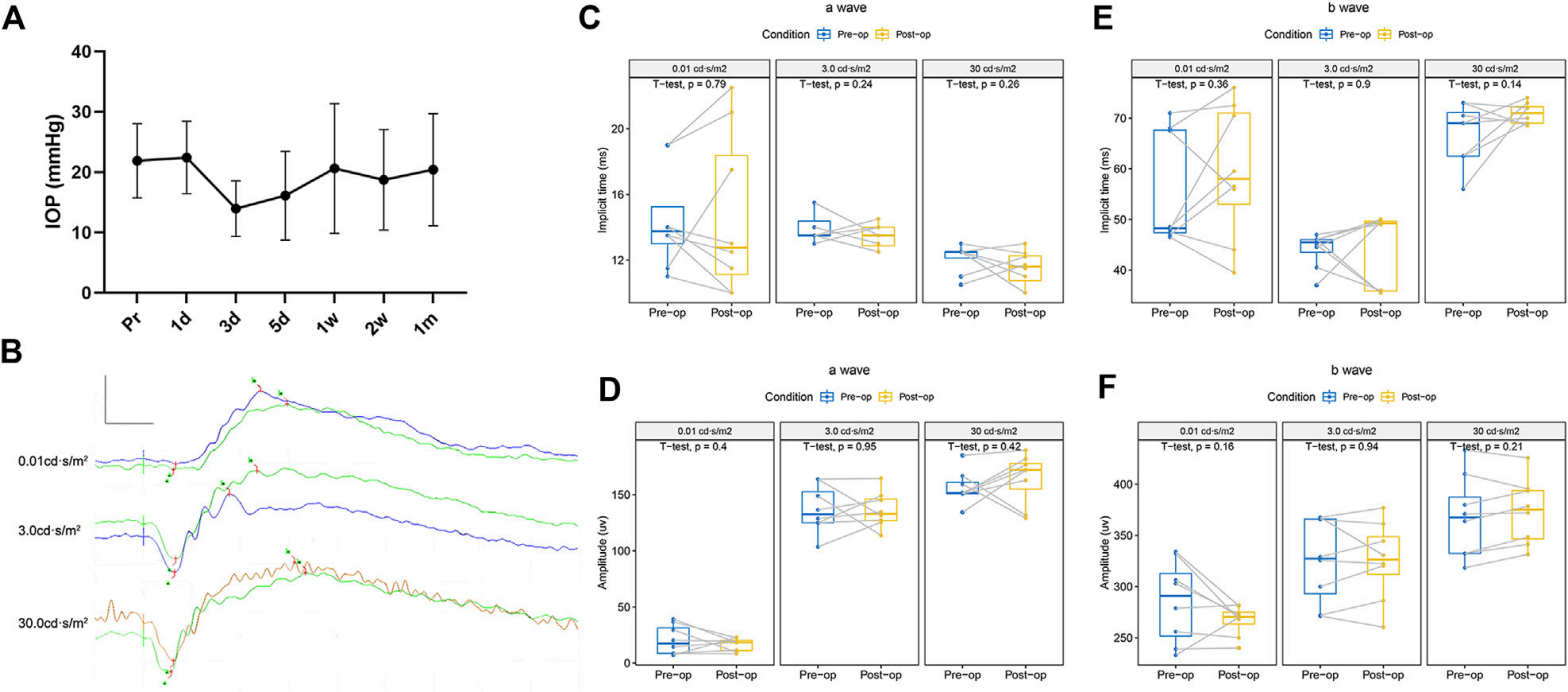
Variation of IOP and function before and 1 month after the operation in the eye with HA-engineered hydrogel coverage. **(A)** The IOP fluctuated within 1 week after the operation and recovered spontaneously within 1 month, with no statistical difference. **(B)** A splicing ERG diagram of the left eye was recorded before (green line) and 1 month after the retina coverage with the HA-engineered hydrogel. Scale bar: 200 uv and 20 ms. **(C–F)** Left eyes’ implicit time and amplitude of the a-wave and b-wave before and 1 month after the retina coverage with the HA-engineered hydrogel.

During the 1-month follow-up, no inflammatory reaction was observed in the eyes on which the HA-engineered hydrogel was applied to the retina. Slit lamp and indirect ophthalmoscopy examinations showed normal conjunctivae, corneas, aqueous humor, crystalline lenses, vitreous humor, and retinas at all time points. No cells or flares were observed in the anterior chamber or vitreous chamber. There was no significant change in IOP of the left eyes pre- and post-operation throughout the observation period (Figure 5A and Supplementary Table S1).

The ERGs of the left eyes before and 1 month after the operation showed the typical components of an ERG, an a-wave followed by a rapidly rising b-wave. Figures 5B–F and Supplementary Table S2 show that there was no significant difference in the pre- and postoperative implicit times and amplitudes of a-waves and b-waves of the ERGs at any stimulus intensity level on the left eyes.

**FIGURE 6:**
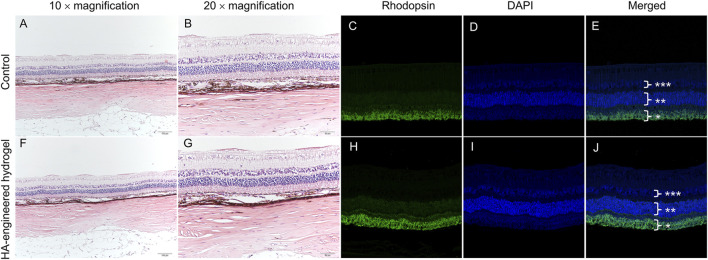
Variation of morphology before and 1 month after the operation in the eye with HA-engineered hydrogel coverage. H&E staining images and immunostaining images of the normal retina **(A–E)** and the retina with HA-engineered hydrogel coverage **(F–J)**. The magnification of immunostaining images was 20 × *, rod photoreceptor layers. **, outer nuclear layer; ***, inner nuclear layer. The scale bar for the 10 × magnification was 100 um and that for the 20 × magnification was 50 um.

Compared with a normal healthy right eye, H&E and immunofluorescence revealed no significant abnormality or inflammation in the left eye, such as the presence of inflammatory cells, epiretinal membrane, retinal edema, disorganization, or atrophic changes of the retinal layers (Figure 6).

### HA-Engineered Hydrogel Significantly Improved the Retinal Reattachment Rates as Retinal Patch in RRD Repairment

**FIGURE 7:**
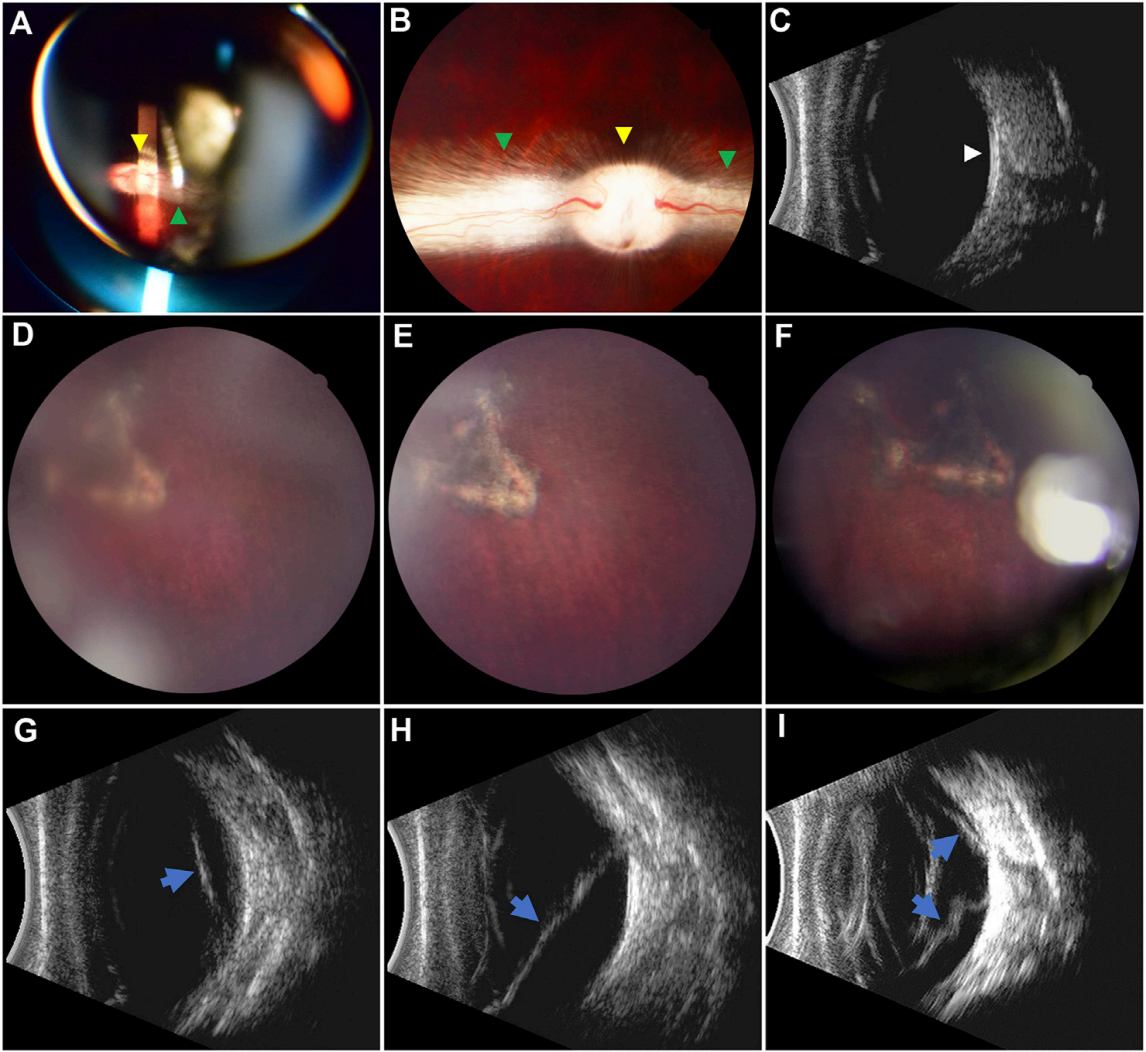
Pre- and postoperative fundus examination. **(A–C)** Preoperative fundus examination *via* indirect ophthalmoscopy, fundus photography, and B-mode ultrasound, respectively. The optic disc (yellow arrowheads) and myelinated nerve fibers (green arrowheads) were well-marked. The vitreous cavity was clear, and the retina was attached (white arrowhead) in the ultrasound image. **(D–F)** Retinal reattachment in the RRD-hydrogel group at 7 days and 1 and 3 months after surgery, respectively. Pigmentation around the retinal break was apparent. **(G–I)** Retinal detachment (blue arrows) in the RRD group at 7 days and 1 and 3 months after surgery, respectively. The localized retinal detachment **(G)** extended **(H)** and gradually became stiff from a soft state over time **(I)**.

There was a significant statistical difference in the retinal reattachment rates between the RRD-hydrogel and RRD groups (100 and 25%, respectively, *p* = 0.007, *χ* ^2^ = 9.6); all retinas were reattached in the eight eyes of the RRD-hydrogel group, while the retina was reattached in two eyes and detached in six eyes in the RRD group throughout the 3-month follow-up. Fundus examination before surgery and typical fundus photography of retinal reattachment in the RRD-hydrogel group and B-mode ultrasound of retinal detachment in the RRD group are shown in Figure 7.

## Discussion

In this study, the HA-engineered hydrogel, a transparent, smooth, and cohesive ophthalmic hydrogel manufactured using a novel linearly cross-linking technology, showed not only its favorable biocompatibility during one-month follow-up, but also high viscosity and cohesiveness as the retinal patch in the RRD model of rabbit eyes during 3-month follow-up.

HA is a naturally occurring linear anionic polysaccharide, which has applications in diverse areas. In clinical ophthalmology, HA has been employed as an artificial tear ingredient for the treatment of dry eyes and a viscoelastic agent for cataract surgery. However, without further modification or functionalization, some inherent disadvantages, such as poor mechanical properties and restricted cell adhesion, have limited its wider application in its natural state. Implementing a cross-linking approach can compensate for these unsatisfactory shortcomings. Cross-linking is a stabilization process in polymer chemistry that results in a network structure depending on a multi-dimensional extension of a chain polymer. By cross-linking, HA is transformed into a gel-like substance that differs from its raw sol-like nature. Hydrogels are classified into two types based on the type of cross-link junctions: physically cross-linked and chemically cross-linked. Physical cross-linking has high biocompatibility and is not toxic, but the junction is reversible *via* hydrogen bonds, hydrophobic interactions, and chain entanglements. On the other hand, chemical cross-linking has irreversible junctions, in which covalent bonds are present between different polymer chains, thus leading to excellent mechanical strength (Lu et al., 2018). The mechanical property, biological function, and degradation behaviors of the hydrogel are strongly dependent on the cross-linker concentration in the chemical cross-linking, for instance, Figure 4D and Supplementary Figure S1 illustrate that the cohesiveness of the HA-engineered hydrogel is similar to that of Healaflow^®^, and much higher than those of Matrifill^®^ and Janlane^®^, though both the cross-linkers are DVS in HA-engineered hydrogel and Matrifill^®^, while it is BDDE in both Healaflow^®^ and Janlane^®^. But the cross-linker may induce cytotoxicity that the human body cannot tolerate when the concentration is too high (Jeon et al., 2007; Lai, 2014). For example, cyanoacrylate is a synthetic gel that provides strong permanent adhesion and continuous seal in seconds in a vast number of experiments and general surgical trials (Guhan et al., 2018). However, localized but definite retinal toxicity in rabbit eyes 1 month after implantation was validated by histological examinations (Hida et al., 1988). Therefore, the evaluation of the biocompatibility of biomaterial is the most important prerequisite before clinical application.

An ideal retinal patch must have certain characteristics on the premise of favorable biocompatibility. First, it must remain firmly adhered and compliant to the retina long enough to allow the development of chorioretinal adhesion induced by classic retinopexy. Second, it should be biodegradable to avoid the need for a second surgical procedure to remove it. Finally, it must be delivered to the eye with minimal or no trauma *via* a simple procedure. The Bio-Alcamid^®^ showed strong adverse effects on the retina in an *in vitro* study, preventing further effectiveness studies (Barth et al., 2014). PEG gel elicited reactions similar to the control retinas in the *in vitro* study. In the *in vivo* study, the PEG gel was not toxic to the eye, could successfully close the retinal breaks, and maintain retinal reattachment. However, the PEG sealant requires the illumination of a xenon arc lamp (450–500 nm, blue-green) for 40–60 s before it can polymerize to form a clear, flexible, and firmly adherent hydrogel (Hoshi et al., 2015; Hoshi et al., 2018). With further technological advancements, the PEG-derived polymer is now stored in powder form, and it needs to be mixed with methylcellulose for 150 s to polymerize before use (Hubschman et al., 2017). The procedure is more complex compared to the injectable properties of Healaflow^®^ and our HA-engineered hydrogel. The commercially available bioresorbable translucent membrane Seprafilm^®^, composed of sodium HA and carboxymethylcellulose, presented favorable biocompatibility and powerful adhesion in the *in vitro* and *in vivo* studies and clinical trials. But the sclerotomy site needs to be expanded to 3 mm to deliver a 5 × 2 mm sheet of Seprafilm^®^ onto the retina (Sueda et al., 2006; Teruya et al., 2009; Haruta et al., 2017). The biological glues, such as representative fibrine glue, are composed of separate glue A and glue B. Retinal breaks must be covered with glue A first, followed by a quick and precise mixture of glue B to form the membrane. Thus, there is a risk of glue migration to the subretinal space due to the fluidity of the glue before membrane formation and the spherical shape of the eyeball(Kwon et al., 2019; Tyagi and Basu, 2019; Wang et al., 2020; Mamlouk et al., 2021). Healaflow^®^ is a slow resorbable and self-degrading viscoelastic agent. In the *in vitro* investigation, it exhibited strong and lasting adhesion, and protection from culture-induced trauma; in the animal experiment, it did not negatively affect retinal morphology or function, indicating favorable biocompatibility; and in a clinical trial, it was found to be beneficial to patch retinal breaks with vitrectomy (Barth et al., 2014; Barth et al., 2016; Ren et al., 2020). According to the review of the current literature on ocular experiments or clinical trials, most adhesives have good biocompatibility and strong tissue adhesiveness, but the manipulation of delivery or covering retinal breaks is complex. The injectable gel property of Healaflow^®^ presents obvious advantages.

Herein, our novel ophthalmic HA-engineered hydrogel is similar to Healaflow^®^ in terms of pH, osmolarity, specific gravity, and refractive index (Table 1), suggesting that the HA-engineered hydrogel exhibits the basic physicochemical properties required as an ideal ophthalmic sealant. In comparison to other tissue adhesives, the HA-engineered hydrogel appears to be a better candidate for use as a retinal patch because its injectable property eliminates the need for expanded sclerotomies for delivery and additional procedures to mix or polymerize. Importantly, the HA-engineered hydrogel displays high dynamic viscosity and cohesiveness, which is similar to Healaflow^®^ (Figure 4). The viscosity and cohesiveness are two curtail properties, which have a close relationship with the biocompatibility and efficacy of the hydrogel sealant. To prevent the migration of RPE cells into the vitreous cavity through retinal breaks, the sealant should possess a high viscosity to ensure its tight adhesion to the surrounded tissues of the breaks (Barth et al., 2016). According to our results, the dynamic viscosity of the HA-engineered hydrogel is similar to that of Healaflow^®^ (252,170 mPa·s for the HA-engineered hydrogel and 258,000 mPa·s for Healaflow^®^ at 0.25 Hz, respectively), both of which are significantly higher than that of PEG (700–1,400 mPa·s) in the previously published reports (Hoshi et al., 2015). Our group has previously verified that Healaflow^®^ could remain adherent for at least 14 days in the culture flask filled with a balanced solution, and could effectively adhere to the retina in RRD patients (Ren et al., 2020). Based on the fact that HA-engineered hydrogel and Healaflow^®^ share almost identical physical and chemical properties, it is assumed that the HA-engineered hydrogel sealant possesses sufficiently high viscosity to meet the need for break sealing. After sealing the breaks, a high cohesiveness keeps the sealant intact and not dissociating under the infiltration of aqueous humor after the sterile air was absorbed in a few days; thus, a prolonged retention time of the sealant and a better clinical effect could be achieved. Our results showed that the HA-engineered hydrogel exhibited similar cohesiveness as compared with Healaflow^®^, and much higher cohesiveness than Matrifill^®^ and Janlane^®^. The discrepancy of cohesiveness was also reflected in the morphology of hydrogels, as the HA-engineered hydrogel and Healaflow^®^ appeared to be smooth and homogeneous, while Matrifill^®^ and Janlane^®^ appeared to be firm and particulate. The high cohesiveness of the HA-engineered hydrogel contributed to a stable and prolonged retinal attachment during the 3-month follow-up period in the animal studies. In addition, the HA-engineered hydrogel exhibited a relatively higher G’ (80–160 Pa at 2% strain under 25°C) than the native vitreous body (1–7 Pa), (Tram et al., 2020), indicating the ability of the HA-engineered hydrogel to rebound to its original shape when acted on by dynamic forces. The G’ of the HA-engineered hydrogel was similar to that of Healaflow^®^, indicating similar resistance to deformation of the two gels.

In the development of any biomaterial, the evaluation of potential inflammatory or toxic responses *in vivo* is critical. The laser-induced chorioretinal adhesion takes about 3 weeks to be strong enough, according to the results of rabbit eyes (Smiddy and Hernandez, 1992), during which time the eyeball is relatively unstable. For instance, the cyanoacrylate tissue adhesive showed retinal toxicity 1 month after the implantation; however, no identifiable distant toxic effects or electrophysiologic changes were observed during the 6-month follow-up period (Hida et al., 1988). Consequently, many studies choose approximately 3 weeks after surgery as the cutoff to report the biocompatibility of tissue adhesives (Sueda et al., 2006; Teruya et al., 2009; Hoshi et al., 2015). In this study, our ERG records, H&E, and immunofluorescence one month after the operation suggested no adverse effects of the HA-engineered hydrogel on retinal function and morphology, confirming its favorable biocompatibility. Simultaneously, our results demonstrated the efficacy of the HA-engineered hydrogel as the retinal patch in the RRD model of rabbit eyes with a 3-month follow-up, which can aid in the complete reattachment of the retina without the need for expansile gas or silicone oil endotamponade. Sterile air as endotamponade combined with the HA-engineered hydrogel as the retinal patch avoids the adverse complications of long-acting gas and silicone oil, discomfort from the strict postoperative face-down position, and the long-term poor visual quality caused by the higher (silicone oil) or lower (long-acting gas) refractive index compared to the natural human vitreous (Hoshi et al., 2019). The IOP fluctuated significantly within 1 week after the operation and recovered spontaneously within 1 month in our study, with no statistical difference. Transient hypotony is a common phenomenon after 25-gauge vitrectomy (Bamonte et al., 2011); thus, the 25-gauge vitrectomy was assumed to be the main risk factor of transient postoperative hypotony in this study.

There are some limitations to this study. First, during the manipulation of the RRD model, the size of the retinal break was small, and the localized retinal detachment only lasted a few seconds; hence, there were few or no RPE cells migrating to the vitreous cavity, and the RRD model failed to fully mimic the pathological processes of human RRD. Second, unlike in human RRD, we did not use endolaser photocoagulation around the retinal break in the RRD model. It is unclear whether the HA-engineered hydrogel eliminates the need for laser retinopexy in clinical trials, despite animal experiments showing 100% retinal reattachment without laser retinopexy. Finally, the behavior of rabbits was uncontrollable during the recovery period.

In conclusion, the HA-engineered hydrogel could play the role of an ophthalmologic sealant *via* its high viscosity and cohesiveness. This pilot study of a small series of RRD models with a short-term follow-up provides preliminary evidence to support the favorable biocompatibility and efficacy of the HA-engineered hydrogel as a promising retinal patch for sealing retinal breaks in retinal detachment repair. More cases and longer follow-up studies are needed to assess the safety and long-term effects of the HA-engineered hydrogel.

## Data Availability

The original contributions presented in the study are included in the article/Supplementary Material; further inquiries can be directed to the corresponding authors.
