# Supplementary material for: Biocompatibility and Efficacy of a Linearly Cross-Linked Sodium Hyaluronic Acid Hydrogel as a Retinal Patch in Rhegmatogenous Retinal Detachment Repairment
Source: Front Bioeng Biotechnol. 2022 Jul 4;10:914675. doi: 10.3389/fbioe.2022.914675 (PMC9289194; doi:10.3389/fbioe.2022.914675)
Supplement: Supplementary file 1 [file Table1.DOCX]

Supplementary Table 1. The variation of IOP in rabbit eyes with HA-engineered hydrogel coverage before and after vitrectomy.

| Time point | IOP（mmHg） | *P* | *χ*^2^ |
| --- | --- | --- | --- |
| Pr | 21.88 ± 6.15 | 0.086 | 11.08 |
| Po 1d | 22.41 ± 6.00 |  |  |
| Po 3d | 13.95 ± 4.59 |  |  |
| Po 5d | 16.10 ± 7.36 |  |  |
| Po 1w | 20.60 ± 10.75 |  |  |
| Po 2w | 18.73 ± 8.31 |  |  |
| Po 1m | 20.40 ± 9.29 |  |  |

IOP: intraocular pressure; Pr: preoperation; Po: postoperation; d: day; w: week; m: month.
