# Supplementary figures and images for: Biocompatibility and Efficacy of a Linearly Cross-Linked Sodium Hyaluronic Acid Hydrogel as a Retinal Patch in Rhegmatogenous Retinal Detachment Repairment
Source: Front Bioeng Biotechnol. 2022 Jul 4;10:914675. doi: 10.3389/fbioe.2022.914675 (PMC9289194; doi:10.3389/fbioe.2022.914675)

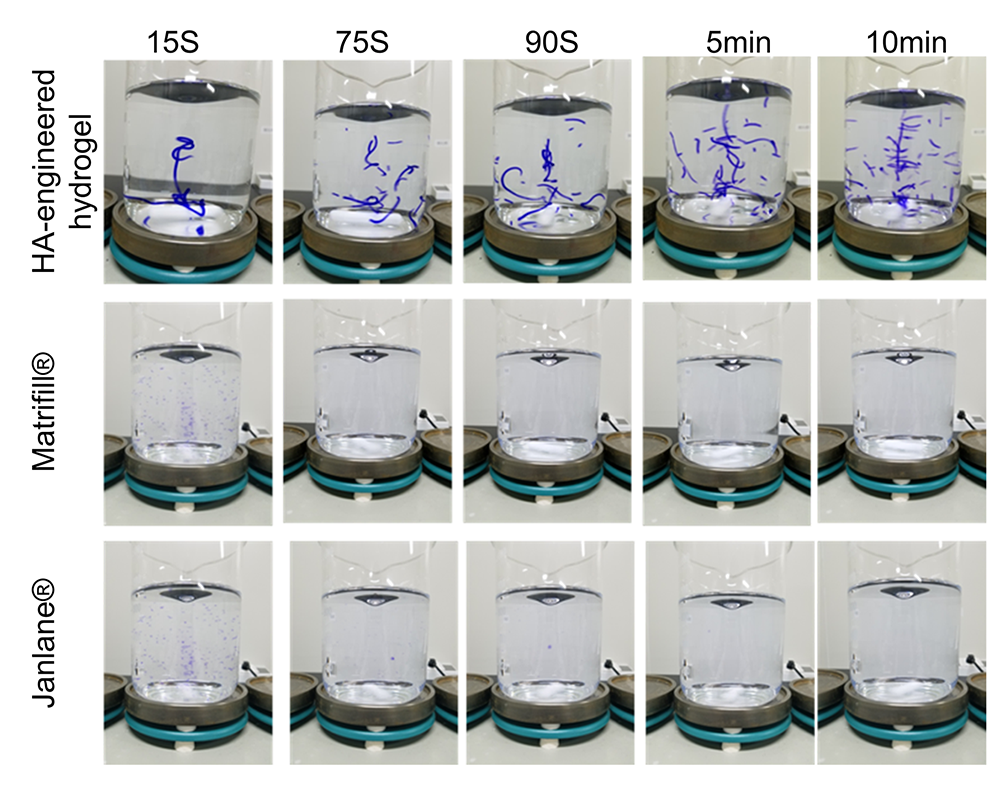

Supplement: Supplementary file 2 [file Image1.TIF]
