# Supplementary material for: Biocompatibility and Efficacy of a Linearly Cross-Linked Sodium Hyaluronic Acid Hydrogel as a Retinal Patch in Rhegmatogenous Retinal Detachment Repairment
Source: Front Bioeng Biotechnol. 2022 Jul 4;10:914675. doi: 10.3389/fbioe.2022.914675 (PMC9289194; doi:10.3389/fbioe.2022.914675)
Supplement: Supplementary file 3 [file Table2.DOCX]

Supplementary Table 2. The implicit time and amplitude of ERG of pre-operation and post-operation in the eyes with HA-engineered hydrogel coverage.

| Implicit time (ms) | | Amplitude (uv) | | No. | Condition | Stimuli |
| --- | --- | --- | --- | --- | --- | --- |
| a wave | b wave | a wave | b wave |  |  |  |
| 11.0 | 47.5 | 8.7 | 256.0 | 1 | Pre-op | 0.01 cd·s/m^2^ |
| 13.5 | 46.5 | 8.6 | 239.0 | 2 |  |  |
| 11.5 | 47.0 | 7.1 | 306.4 | 3 |  |  |
| 14.0 | 48.0 | 14.6 | 332.5 | 4 |  |  |
| 13.5 | 48.5 | 20.3 | 303.0 | 5 |  |  |
| 14.0 | 68.0 | 29.6 | 233.1 | 6 |  |  |
| 19.0 | 67.5 | 39.2 | 278.9 | 7 |  |  |
| 19.0 | 71.0 | 36.8 | 334.1 | 8 |  |  |
| 10.0 | 44.0 | 11.6 | 250.1 | 1 | Post-op |  |
| 10.0 | 39.5 | 8.4 | 240.1 | 2 |  |  |
| 17.5 | 56.5 | 22.9 | 271.1 | 3 |  |  |
| 13.0 | 70.5 | 18.8 | 268.1 | 4 |  |  |
| 12.5 | 59.5 | 19.5 | 273.1 | 5 |  |  |
| 11.5 | 76.0 | 9.8 | 270.0 | 6 |  |  |
| 22.5 | 56.0 | 18.4 | 281.9 | 7 |  |  |
| 21.0 | 72.5 | 22.6 | 281.3 | 8 |  |  |
| 13.5 | 46.0 | 163.8 | 367.6 | 1 | Pre-op | 3.0 cd·s/m^2^ |
| 15.5 | 40.5 | 124.9 | 300.0 | 2 |  |  |
| 14.0 | 45.0 | 136.5 | 325.7 | 3 |  |  |
| 13.0 | 46.0 | 125.1 | 329.0 | 4 |  |  |
| 13.5 | 46.0 | 163.8 | 367.6 | 5 |  |  |
| 13.5 | 47.0 | 148.9 | 365.5 | 6 |  |  |
| 13.5 | 37.0 | 128.8 | 271.3 | 7 |  |  |
| 15.5 | 44.5 | 103.6 | 272.1 | 8 |  |  |
| 14.0 | 35.5 | 164.6 | 330.7 | 1 | Post-op |  |
| 13.5 | 36.0 | 149.1 | 320.4 | 2 |  |  |
| 13.0 | 35.5 | 145.2 | 322.3 | 3 |  |  |
| 14.0 | 50.0 | 127.4 | 344.5 | 4 |  |  |
| 14.5 | 49.5 | 131.4 | 376.9 | 5 |  |  |
| 12.5 | 49.0 | 113.8 | 361.4 | 6 |  |  |
| 12.5 | 49.5 | 134.7 | 260.4 | 7 |  |  |
| 13.5 | 50.0 | 125.4 | 286.4 | 8 |  |  |
| 12.5 | 73.0 | 150.8 | 332.4 | 1 | Pre-op | 30 cd·s/m^2^ |
| 11.0 | 69.0 | 166.7 | 433.3 | 2 |  |  |
| 12.5 | 62.5 | 159.3 | 410.0 | 3 |  |  |
| 12.5 | 62.5 | 134.4 | 364.0 | 4 |  |  |
| 12.5 | 56.0 | 185.0 | 371.0 | 5 |  |  |
| 13.0 | 70.5 | 151.3 | 318.4 | 6 |  |  |
| 12.5 | 73.0 | 150.8 | 332.4 | 7 |  |  |
| 10.5 | 69.0 | 151.8 | 379.9 | 8 |  |  |
| 10.0 | 72.0 | 182.0 | 341.3 | 1 | Post-op |  |
| 12.2 | 74.0 | 131.7 | 425.8 | 2 |  |  |
| 11.0 | 73.0 | 129.2 | 395.4 | 3 |  |  |
| 13.0 | 69.0 | 171.8 | 378.5 | 4 |  |  |
| 11.5 | 72.0 | 189.7 | 372.1 | 5 |  |  |
| 12.4 | 69.0 | 172.2 | 331.5 | 6 |  |  |
| 10.0 | 68.5 | 162.8 | 348.4 | 7 |  |  |
| 11.7 | 70.0 | 176.4 | 393.4 | 8 |  |  |
